# An ultra-high frequency radio frequency identification system for studying individual feeding and drinking behaviors of group-housed broilers

**DOI:** 10.1017/S1751731118003440

**Published:** 2019-01-11

**Authors:** G. Li, Y. Zhao, R. Hailey, N. Zhang, Y. Liang, J. L. Purswell

**Affiliations:** 1 Department of Agricultural and Biological Engineering, Mississippi State University, Mississippi State, MS 39762, USA; 2 Rural Energy Research Institute, Heilongjiang Academy of Agricultural Sciences, Harbin, Heilongjiang 150086, China; 3 Department of Electrical and Computer Engineering, Mississippi State University, Mississippi State, MS 39762, USA; 4 USDA-ARS, Poultry Research Unit, Mississippi State, MS 39762, USA

**Keywords:** poultry, behavioral detection, system setup, validation

## Abstract

Radio frequency identification (RFID) technology offers a real-time solution to monitor behavioral responses of individual animals to various stimuli, which provides crucial implications on farm management and animal well-being. The objectives of this study were to (1) develop and describe an ultra-high frequency radio frequency identification (UHF-RFID) system for continuously monitoring feeding and drinking behaviors of individual broilers in group settings; and (2) validate the performance of the UHF-RFID system against video analysis in determining the instantaneous bird number (IBN) and time spent (TS) at feeder and drinker. The UHF-RFID system consisted of cable-tie tags, antennas, a reader and a data acquisition (DAQ) system. The antennas generated electromagnetic fields where tags were detected and registered by the DAQ system. Electromagnetic fields of the antennas were modified to cover areas of concern (i.e. tube feeders and nipple drinkers) through a series of system evaluations and customizations including tag sensitivity test, power adjustment, radio wave shielding, and assessment of interference by add-ons (e.g. plastic wraps for protecting antennas and an empty carton box for zoning out broilers) and feed/feeder. System validation was performed in two experimental rooms, each with 60 tagged broilers. The results showed that the max reading distances of tags with an identical manufacturer’s specification were markedly different, indicating large variations in sensitivity among the tags. Desired electromagnetic fields could be achieved by adjusting the power supplied to antennas and by partially shielding antennas with customized stainless steel sheets. The protection materials and fully loaded feeder had little effect on electromagnetic fields of the antennas. The accuracies of the UHF-RFID system for determining IBN and TS were, respectively, 92.5±4.2% and 99.0±1.2% by the feeder antennas and 94.7±4.2% and 93.7±6.9% by the drinker antennas. It is concluded that the UHF-RIFD system can accurately detect and record feeding and drinking behaviors of individual broilers in group settings and thus is a useful tool for investigating impacts of resource allocations and management practices on these behaviors.

## Implications

The customized ultra-high frequency radio frequency identification (UHF-RFID) system can be used to track the feeding and drinking behaviors of individual broilers. The feeding and drinking behaviors of broilers can be used as indicators of bird health, resource utilization, and productivity problems, thus have critical welfare and economic implications on broiler production. The system customizations and validations presented in this study demonstrated standard procedures to improve accuracy of UHF-RFID systems for broiler behavioral monitoring.

## Introduction

Assessments of poultry feeding and drinking behaviors help to understand bird utilization of feed and water resources, thus have critical economic and welfare implications for poultry industry (Gonyou, 1994; Prayitno *et al*., 1997). Previous studies have investigated poultry feeding and drinking behaviors as affected by management practices (Savory and Mann, 1999), environmental stimuli (May and Lott, 1994), and rearing systems (Tanaka and Hurnik, 1992). Of these studies, many recorded poultry behaviors through manual observation, e.g. identifying birds and behaviors visually by investigators. Manual observation is suitable for behavioral studies with small sample sizes; however, it becomes laborious and impractical as the sample size increases and multiple behavioral responses are required to be monitored simultaneously. Development of automatic systems that can accommodate large sample sizes and monitor multiple behaviors is warranted.

Some automatic monitoring systems have been developed for studying group-housed poultry behaviors. For example, weighing scale systems were used to monitor real-time feed and water consumptions (Lott *et al*., 1992; Puma *et al*., 2001). While these systems successfully recorded the feed and water uses of the entire group, they were not capable of monitoring behaviors of individual birds, therefore missing the information of individual variations within a group.

Radio frequency identification offers a solution to simultaneously monitor behaviors of multiple individual animals by registering the tagged animals entering electromagnetic fields of antennas (Maselyne *et al*., 2014; Sales *et al*., 2015). The commercially available antennas vary in size and shape and can be incorporated into existing animal production systems with proper modifications. Radio frequency identification systems have been used for automatically monitoring feeding and drinking behaviors of swine, turkeys, and laying hens, and have demonstrated high accuracy of sampling (Tu *et al*., 2011; Brown-Brandl *et al*., 2013; Maselyne *et al*., 2016; Li *et al*., 2017). Nakarmi *et al*. (2014) designed a RFID matrix and algorithms to track trajectory movements of individual laying hens, and register their feeding, drinking, perching and nesting behaviors.

Although RFID system has been employed for monitoring behaviors of many domestic animal species it has not been developed for use in group-housed broilers in the open flooring housing system which is typical practice at US broiler commercial farms. The objectives of this study were to: (1) develop a UHF-RFID system for continuously monitoring feeding and drinking behaviors of individual broilers in group settings, and (2) validate the UHF-RFID system against video analysis in determining the instantaneous bird number (IBN) and time spent (TS) at feeder and drinker.

## Material and methods

### Ultra-high frequency radio frequency identification system

The UHF-RFID system consisted of four elements: tags, antennas, a reader and a data acquisition (DAQ) system (Supplementary Figure S1). The antennas generated electromagnetic fields that registered uniquely coded RFID tags and the reader (IPJ-REV-420; TransTech Systems Inc., Wilsonville, OR, USA) subsequently transmitted IDs of the tags to the DAQ system. In animal tests, the tags were attached to the necks of birds (Supplementary Figure S2), and antennas were placed closely to the areas of concern (i.e. underneath tube feeders and next to nipple drinkers). Cable-tie tags (PT-103; TransTech Systems Inc., Wilsonville, OR, USA) were used in this study because they were small and could be easily attached to birds (Oliveira *et al*., 2016). A square antenna (TIMES-7 A6034S; Impinj Inc., Seattle, WA, USA) and a rectangular antenna (IPJ-A0303-000; Impinj Inc., Seattle, WA, USA) were selected to register feeding birds at the tube feeder and drinking birds at the nipple drinker, respectively. The UHF-RFID tags were manufactured by Technologies ROI LLC (SC, USA).

### System performance tests

#### Variations in tag sensitivity

**Figure 1:**
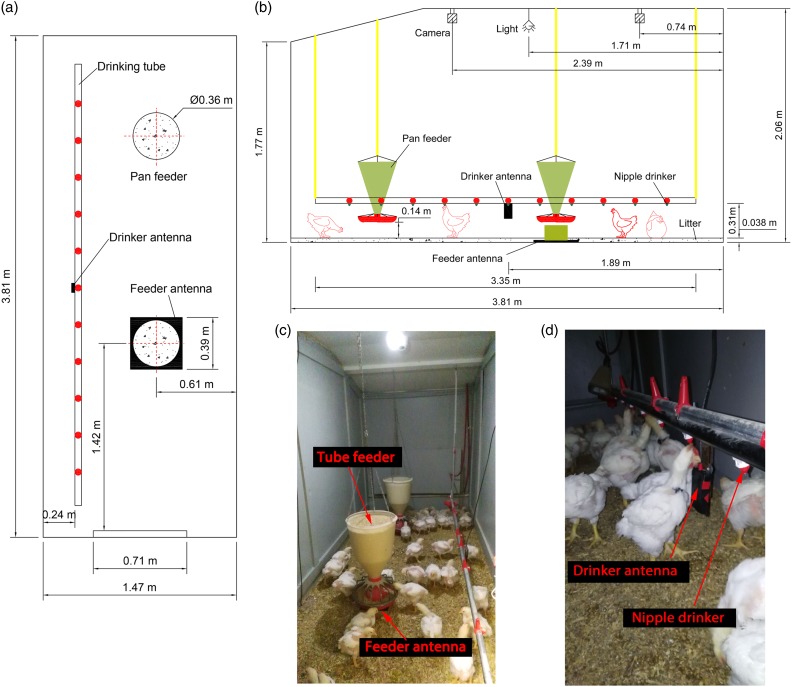
Schematic drawings and photos of the experimental room and antennas for the behavioral study of Ross×Ross 708 broilers: (a) top view; (b) side view; (c) a photo for the placement of the feeder antenna; (d) a photo for the placement of the drinker antenna.

Cable-tie tags with the same manufacture specifications may be excited at different electromagnetic strengths or, in other words, be registered at different distances from an antenna. To understand the variations among tags and select those with similar sensitivities for the animal test, max reading distances (MRD) of cable-tie tags from a square antenna (TIMES-7 A6034S; Impinj Inc., Seattle, WA, USA) were determined using the procedures described below. The antenna was horizontally placed and provided with the power of 0.2 W. A cable-tie tag was placed at the center of the testing antenna. The tag was then moved perpendicularly up from the antenna until it could no longer be detected. At this moment, the distance between the tag and the antenna was recorded as the MRD. At the MRD, the tag was rotated in the horizontal plane to make sure a true MRD. The way of tag positioning and rotating in this test simulated how a tagged bird approached to the feeder or drinker antenna (Figure 1c and 1d). The position and movement of tags were applied for the entire system performance tests. In this session, the MRDs of tags at the center of the antenna were determined. Basic descriptive statistical methods (e.g. mean, standard deviation, and CV) were calculated to evaluate the variations among tags. To select tags with similar sensitivities for the animal test (or remove the outliers), a method of inter-quartile range (IQR) was used (Tukey, 1977). The first quartile (Q_25_) was the 25th percentile of the MRD data, and the third quartile (Q_75_) was the 75th percentile of the MRD data. The IQR was defined as the difference between Q_25_ and Q_75_. The lower and upper inner fences were defined by Q_25_-(1.5×IQR) and Q_75_+(1.5×IQR), respectively. The MRD data out of the lower and upper inner fences were treated as outliers. Tags with MRD data located within the inner fence were deemed similar in sensitivities and used in the animal test. To observe the MRD difference of same tags, five additional tags (tag IDs: A359, A362, A372, A384, and A393) were randomly selected and the MRD of each of these tags was measured for three times. The MRDs of these tags held by wooden sticks were compared with those held by a hand to check the hand effect on the system performance tests. The MRDs of these five tags were tested when the tags were leaving and entering the electromagnetic field of the antenna. Due to the variations in tags, four tags with similar sensitivities were selected via the IQR method mentioned above and used for the following system performance tests.

#### Shielding effect of steel sheets on electromagnetic field of the feeder antenna

The system setup could be found in Figure 1. The square antenna was larger than the tube feeder (Supplementary Figure S3), which produced areas of unconcern. To create an electromagnetic field at the space of interest directly above the feeder pan, radio waves emitted from corners of the square antenna needed to be shielded. Because stainless steel sheets can effectively block the wave emission from antennas (Sales *et al*., 2015), 1.5-mm-thick stainless steel sheets with different sizes of center openings were fabricated and their shielding effects on the electromagnetic field at the corners of the feeder antenna were investigated. The tested steel sheets had the same dimension (40×40 cm, L×W) as the antenna, but differed in the diameters (46, 43, 41, 38, and 36 cm) of the center openings (Supplementary Table S1). They were bolted to the feeder antenna during the test. Based on the results of section ‘Variations in tag sensitivity’, four tags with similar sensitivities in the acceptable MRD range were randomly selected for this test. The four tags (each measured for three times and the same in the following) were used to test the MRD at the shielded corners of the feeder antenna at four power settings (Supplementary Table S1). The tags were held for 3 to 5 s at the MRD for each measurement. The steel sheets with all opening sizes were initially tested at 1.0 W – the maximal power setting. The steel sheet with a 36-cm-diameter center opening shielded the electromagnetic field most effectively and appropriately, therefore its shielding effect was fine-tuned at other power settings of 0.8, 0.6 and 0.5 W.

#### Effect of other add-ons and feed/feeder on electromagnetic field

In the animal test, the feeder antenna was protected with plastic wraps and placed on the litter floor and ~14 cm below the hanging feeder. An empty carton box was placed in the gap between the antenna and the feeder to prevent birds staying underneath the feeder (Supplementary Figure S4a). The effects of one layer of plastic wrap, an empty carton box (21.5×20.6×17.4 cm, L×W×H), and a fully loaded feeder on the electromagnetic field of a shielded antenna were investigated in four scenarios (Supplementary Table S2). Supplying power of the antenna was set to 1.0 W. Max reading distances of four tags were determined at the three different points near the center of the antenna for each scenario. Plastic wraps were also used to protect the drinker antenna, and MRDs of four tags above the drinker antenna with or without plastic wraps were determined through the same method as that for the feeder antenna.

#### Electromagnetic fields of antennas

Electromagnetic fields of the feeder and drinker antennas were measured with all add-ons and a fully loaded feeder, which simulated the system setup in the validation tests. For the feeder antenna, the MRDs of four tags were determined at 53 points above the antenna (Supplementary Figure S4). For the drinker antenna, the MRDs of four tags at 17 points above the antenna were determined (Supplementary Figure S5). The power settings were 0.8 W for the feeder antenna and 1.0 W for the drinker antenna. The MRD data were interpolated to produce electromagnetic fields of the antennas in a 3D coordination using Matlab (R2014b; MathWorks, Natick, MA, USA). In addition to the testing points indicated in Supplementary Figures S4 and S5, some extra fields beyond the borders of both antennas were also tested. However, the signal in those fields was weak (MRD was ~1 to 2 cm) and unstable. Therefore, only the fields right above both antennas were presented in this study.

### Validation of the ultra-high frequency radio frequency identification system

#### Housing, animal and management

Two experimental rooms were used for the UHF-RFID system validation, and each measured 3.81 m long, 1.47 m wide, and 2.06 m high (Figure 1). Each room was equipped with two 36-cm-diameter tube feeders and 11 nipple drinkers. Pine shavings (~4-cm thick) were used as the bedding material. A dimmable LED light bulb was installed at the center of the ceiling. Light intensity at bird level was adjusted according to typical practices at commercial broiler farms. In each room, the wrapped square antenna was placed on the litter floor below one of the suspending tube feeders. An empty carton box was placed in the gap between the feeder and the antenna. A drinker antenna was attached to a bracket mounted to one of the nipple drinkers. See Figure 1 for details of the system setup. All procedures were approved by the USDA-ARS Institutional Animal Care and Use Committee at Mississippi State.

One hundred and twenty 28-day-old Ross×Ross 708 broilers were equally allocated to the two experimental rooms (60 birds each). A tag was tied, like a collar, to the neck of each bird (Supplementary Figure S2). Only tags within the acceptable MRD range based on IQR method were used for the test. To minimize discomfort, the tag collar was loose enough to tuck in an index finger. The broilers were inspected on a daily basis. The size of the collar was regularly adjusted to avoid discomfort (e.g. panting, choking, etc.). Standard broiler diets were supplied. Water and feed were provided *ad libitum*. Lighting schedule was 16-h lighting and 8-h darkness (ON at 0500 h and OFF at 2100 h). During the test period of the experiment, the daily temperature and relative humidity at bird level were maintained at 20.7±2.0°C and 64±6% (mean±SD), respectively.

#### Data collection

The UHF-RFID system continuously registered broilers at feeder and drinker. The data were exported as the text documents (.txt) and then processed in Excel using Visual Basic for Applications. Broiler behaviors at the tube feeder and nipple drinker with antennas were videotaped using two high-definition cameras (Vandal Proof IR Dome Camera; Backstreet 248 Surveillance, LLC., Salt Lake, UT, USA) installed at the ceiling of each experimental room. The video files (2 frame per second, or 2 fps) were saved as AVI format in the network video recorder. All frames of the video files were extracted using Free Video to JPG Converter (ver. 5.0). Numbers of broilers in these frames were manually counted and compared with the corresponding data of the UHF-RFID system.

#### Duration of intermittent withdrawal from a feeder/drinker in a continuous feeding/drinking event

In a continuous feeding/drinking event, a bird could shortly withdraw from the feeder/drinker for swallowing based on manual observation (Li *et al.*, 2017). This yielded reading gaps in the continuous feeding/drinking events. The duration of intermittent reading gaps was determined via a histogram analysis of feeding and drinking events in 10 video episodes (Li *et al*., 2017). The time gaps between two adjacent readings of 10 individual birds were determined. Then a histogram of the time gaps was generated and analyzed. A duration that yielded 95% coverage of the RFID readings in the histogram was used to fill the time gaps.

#### System validation

The broilers at feeder and drinker detected by the UHF-RFID system were compared to those observed manually in the images. A broiler was identified as ‘at feeder/drinker’ when it was eating/drinking, or when it stood at the feeder/drinker and its head directed to the feeder/drinker. The validation tests of drinking behaviors were performed with three different antenna placements (i.e. vertical placement at 23-cm height, vertical placement at 18-cm height, and tilting placement at 18-cm height) (Supplementary Figure S6). Each placement was tested for 2 days. As we wanted to spread the validation periods within and across days, rather than focusing on a specific day and time, 2 min of every 2 h (0600, 0800, 1000, 1200, 1400, 1600, 1800, and 2000 h) in the 16 lighting hours were selected. Total 55 2-min videos were collected in a 7-day period (in the 7^th^ day, seven videos were selected for the validation). These videos were then converted to 13200 frames of images for the validation. Li *et al*. (2018) also reported that broilers spent average 1.3 to 2.0 min for single feeder visit registered by the UHF-RFID system. Therefore, 2-min episodes were enough to cover these behaviors and the behavior transit for validation purposes. In the 2-min periods, the IDs of feeding and drinking birds recorded by RFID system at 1-s intervals were summarized. Based on those information, we determined the IBN at feeder/drinker in each second and the overall TS at feeder/ drinker of each bird. These RFID data were compared with the visual observation data for determining the accuracy. It did not affect the validation at all if some birds already started feeding/drinking at the beginning of the 2 min or did not complete feeding/drinking at the end of the 2 min.

The equations for calculating the accuracy are shown as follows:

(1) For IBN:
(1)
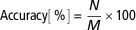

where *N* is number of seconds when RFID could register correct number of birds at feeder/drinker. *M* is the total number of seconds in 2 min.
(2) For TS
(2)
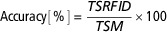

where *TSRFID* and *TSM* are the TS detected by the RFID system and by human observation in 2-min videos, respectively. Time spent is the sum of bird number at feeder/drinker in 2-min videos.

#### Example continuous behavioral monitoring

Numbers of birds at a feeder (out of two feeders) and at a drinker (out of 11 drinkers) were presented from 0912 to 1012 at 35 days of bird age. These results indicated that the system could continuously monitor group-reared birds at feeder/drinker. To elaborate that the system could also continuously monitor the individual behaviors, seven randomly selected birds with unique IDs (A005, A007, A012, A021, A023, A029, and A045) at feeder and the other seven birds (A002, A028, A034, A049, A050, A057, and A088) at drinker were also presented within the same hour.

### Statistical analysis of results

One-way ANOVA with LSD *posthoc* analysis was used to compare the MRDs of tags above the antennas as affected by a hand and wooden sticks, leaving and entering the electromagnetic field, center opening sizes of the steel sheets, supplying powers, add-ons and feed/feeder, in Statistical Analysis Software (SAS 9.3; SAS Institute Inc.). All data were analyzed using PROC GLM statement and more details about the statistical model are described in Supplementary Material S1. The effects were considered significant at a probability level of 0.05. The root mean square error (RMSE) was also provided to quantify the differences between values predicted by the model and the values observed. A sample code for data analysis is provided in Supplementary Material S2.

## Results

### Variations in tag sensitivity

**Figure 2:**
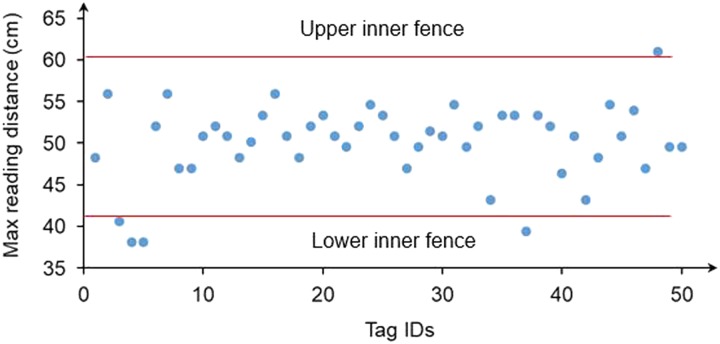
The max reading distances of 50 cable-tie tags above an antenna at power of 0.2W.

Max reading distances of 50 cable-tie tags were determined above the center of a square antenna. The mean±SD of MRD was 50.1±4.6 cm (Figure 2). The first quartile (Q_25_), the third quartile (Q_75_), the 1.5×IQR, the upper inner fence and the lower inner fence were 48.3, 53.3, 7.5, 61.0 and 40.6 cm, respectively. In Figure 2, one tag ((48, 60.1), tag ID and MRD) is above upper inner fence and four tags ((3, 40.5), (4, 38.1), (5, 38.1) and (37, 39.3)) are below lower inner fence. These tags were treated as outliers. After excluding these tags, the mean of MRD±SD was 50.8±3.0 cm with a CV of 5.9%. Max reading distances of five extra tags held by a hand were 64.0±1.0 cm for A359, 55.7±1.5 cm for A362, 54.3±1.5 cm for A372, 46.7±1.5 cm for A384, and 47.7±1.5 cm for A393. Max reading distances of these tags held by wooden sticks were 67.3±0.6 cm for A359, 55.0±1.7 cm for A362, 58.0±1.0 cm for A372, 47.0±1.0 cm for A384, 46.7±1.2 cm for A393. The mean MRDs of these five tags held by a hand and wooden sticks were 53.7±6.6 and 54.8±8.0 cm (*P* = 0.25, RMSE = 0.10). The mean MRDs of these five tags leaving and entering the field were 52.8±7.8 and 53.2±8.1 cm, respectively (*P* = 0.33, RMSE = 0.10).

### Shielding effect of steel sheet on electromagnetic field

**Figure 3:**
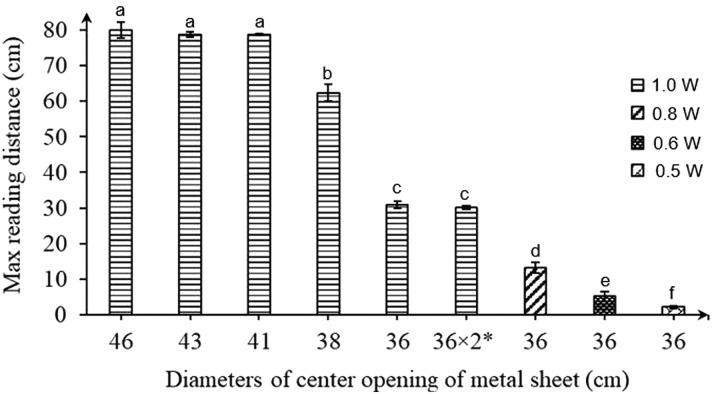
Arithmetical mean maximum reading distances of radio frequency identification tags at corners of the feeder antenna shielded by stainless steel sheets with different opening sizes at four power settings. *Two layers of steel sheets with 36-cm-diameter center openings. Means with different letters on the top of bars are significantly different at *P* < 0.05 (*n* = 4).

Figure 3 shows the mean MRDs of four tags at the shielded corners of the feeder antenna covered by the stainless steel sheets with different opening sizes at four power settings. At 1.0 W, the MRDs were similar when the antenna was covered by sheets with center opening sizes of 41 to 46 cm. The MRDs were significantly reduced for opening sizes of 36 and 38 cm, indicating better shielding effects by these sheets. An extra layer of an identical sheet to the existing one with a 36-cm-diameter opening provided no additional shielding effect on radio waves, because the MRDs were similar (31.1±0.9 cm for one layer *v.* 30.5±0.3 cm for two layers). Reducing the input power to 0.8 W was an effective way to minimize the MRD to 13.5±1.3 cm for the sheet with a 36-cm-diameter opening. At the power of 0.6 and 0.5 W, the MRDs were further reduced (5.1±1.1 and 2.3±0.2 cm, respectively). Supplementary Table S3 shows the *P*-value and RMSE are <0.0001 and 0.46, respectively. Based on our field measurements, the average height of tags attached to 28-day-old standing broilers was ~18 cm. In this study, power was set to 0.8 W and one layer of metal sheet with a 36-cm-diameter center opening was used in the animal tests. In this section, the signal shielding at the corners was the main objective. It was not necessary to test the full grid because only electromagnetic field at the corners need to be blocked to avoid registering birds nearby the feeder without eating. The full-grid electromagnetic fields of shielded antennas were determined in a latter step.

### Effect of other add-ons and feed/feeder on electromagnetic field

**Figure 4:**
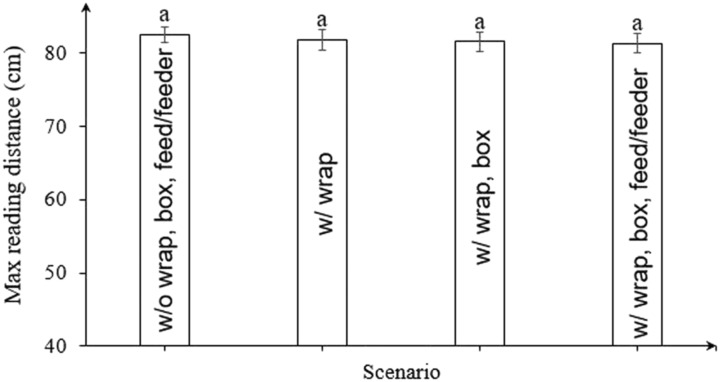
The maximum reading distances of radio frequency identification tags above a feeder antenna with or without protective plastic wraps, a carton box and feed/feeder at power of 1.0 W. Means with the different letters on the top of bars are significantly different at *P* < 0.05 (*n* = 4). ‘w/o’ and ‘w/’ in the figure mean ‘without’ and ‘with’, respectively.

Figure 4 shows the MRDs of tags above the feeder antenna with and without plastic wraps, carton box and feed/feeder. The mean MRDs±SD were 82.3±0.9 cm for the feeder antenna without all add-ons and feed/feeder, 81.9±1.2 cm for the antenna with protective plastic wraps, 81.5±2.5 cm for the antenna with wraps and a carton box, and 81.3±1.2 cm for the antenna with all add-ons and a fully loaded feeder. Supplementary Table S4 shows the *P*-value and the RMSE are 0.47 and 0.25, respectively. The mean MRDs numerically reduced as more add-ons were used, but the difference was not statistically significant. Supplementary Table S5 shows the mean MRD of tags above the drinker antenna with and without plastic wraps are 6.3±2.4 and 6.1±1.5 cm, respectively, and the difference is not significant (*P* = 0.34, RMSE = 0.19). The results indicated little effect on electromagnetic field of the antennas by these add-ons and feed/feeder. Based on the observation, the box worked fine during the one-week test. We did not test for an extended period, but there should be other simple alternatives if the carton box would not last long. In this section, the interference effect of radio waves by the add-ons and feed/feeder was the main concern. It was enough to select a few representative testing points for this objective. Therefore, three different points near the center of the antennas were selected for the test. Electromagnetic fields of the shielded antennas were determined in a latter step.

### Electromagnetic field of antennas

**Figure 5:**
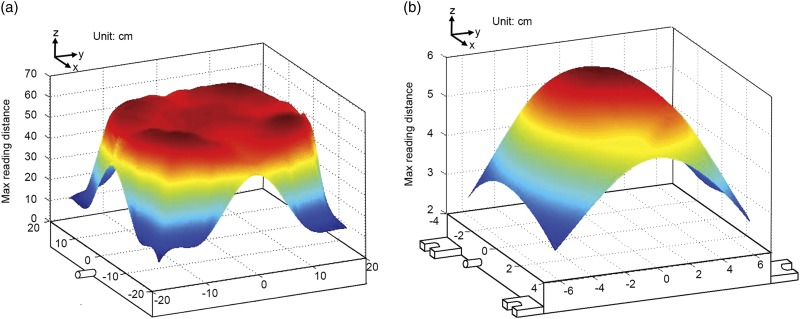
Electromagnetic fields of (a) a feeder antenna (with one-layer stainless steel sheet with a 36-cm-diameter center opening, plastic wraps, a carton box and a fully loaded feeder at 0.8 W), and (b) a drinker antenna (with protective plastic wraps at 1 W).

With the same setup as in the validation tests, electromagnetic fields of feeder and drinker antennas are delineated in Figure 5. Each point at the colorful dome surface in Figure 5 represents the measured or interpolated MRD of RFID tags above the corresponding projection point of an antenna placed in x-y plane. For the feeder antenna, the MRDs were 51.2±5.2 cm for the center area and 12.6±1.3 cm for the corners, respectively (Figure 5a). Such electromagnetic field is reasonable to register the eating broilers and ignore those walking by the feeder without eating. The MRD at the center of the drinker antenna was 6.2±0.3 cm (Figure 5b). However, the mean MRD at the corners of the drinking antenna was 2.5±0.3 cm, which could not be sufficient to register a drinking broiler. The result implies the need for strategic placements (e.g. the tilting placement) of drinker antennas in order to maximize the chance for registering drinking broilers.

### Duration of intermittent withdrawal from a feeder/drinker in a continuous feeding/drinking event

**Figure 6:**
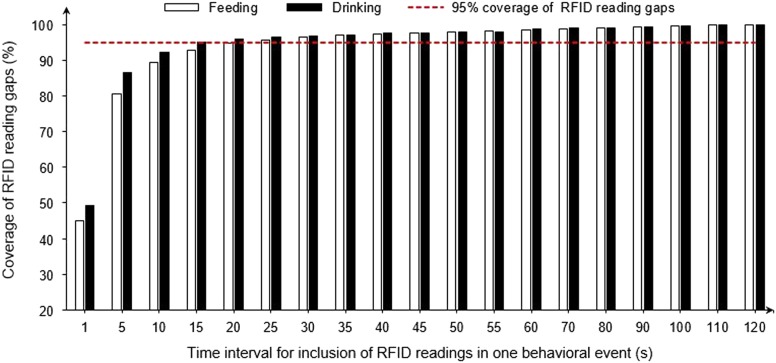
Coverage of radio frequency identification (RFID) reading gaps *v*. time interval for including two gapped RFID readings in one behavioral event for Ross×Ross 708 broilers.

Figure 6 shows coverage of RFID reading gaps at different intervals for including two gapped RFID readings in one behavioral event. A threshold of 20 s for inclusion of RFID data in a single feeding/drinking behavior provided, respectively, 94.7% and 96.0% coverage of the data collected by the UHF-RFID system. In other words, ~95% of the intermittent withdrawal (or swallowing) behaviors lasted less than 20 s.

### Accuracy of the ultra-high frequency radio frequency identification system

The UHF-RFID system was accurate in monitoring broiler feeding behaviors. The mean accuracies were 92.5% for IBN and 99.0% for TS at feeder (Table 1). The accuracy of the UHF-RFID system for drinking behaviors was affected by placements of the drinker antenna. Specifically, the mean accuracies of IBN and TS at drinker were, respectively, 77.2% and 68.4% for the vertical placement at 23 cm height, 89.8% and 73.1% for the vertical placement at 18 cm height, and 94.7% and 93.7% for the tilting placement at 18 cm height.

**Table 1:** Accuracy of the UHF-RFID system (relative to visual observation) for monitoring instantaneous bird number (IBN) and time spent (TS) at feeder and drinker in terms of Ross×Ross 708 broilers

	Accuracy (mean±SD (%))	
Behaviors	IBN	TS
Feeding	92.5±4.2	99.0±1.2
Drinking		
Vertical placement at 23 cm height	77.2±19.5	68.4±21.3
Vertical placement at 18 cm height	89.8±10.8	73.1±27.3
Tilted placement at 18 cm height	94.7±4.2	93.7±6.9

UHF-RFID = ultra-high frequency radio frequency identification.

### Continuous behavioral monitoring by the ultra-high frequency radio frequency identification system

**Figure 7:**
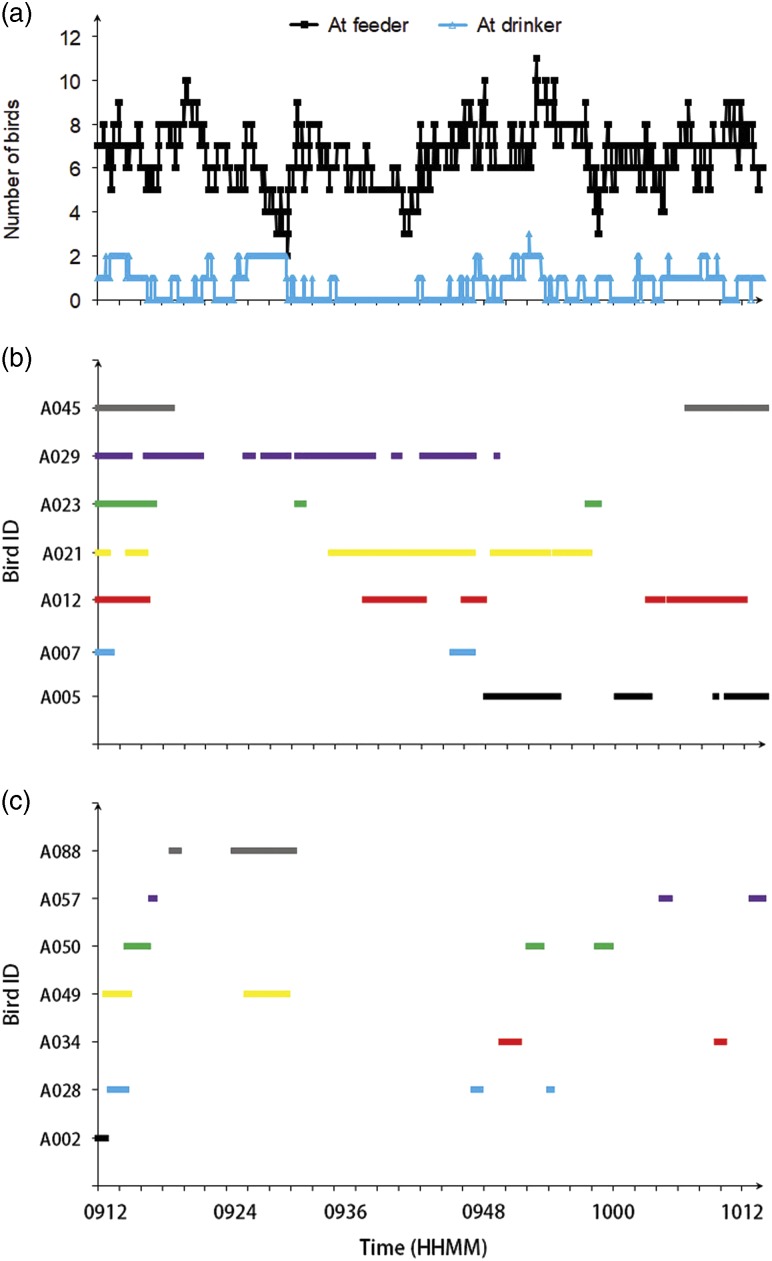
Example continuous behavioral monitoring from 0912 to 1012 on 35 days of age for Ross×Ross 708 broilers: (a) the number of birds out of 60 testing birds at feeder and drinker; (b) seven randomly selected birds at feeder; (c) seven randomly selected birds at drinker.

Figure 7a shows the number of broilers at one tube feeder (out of two feeders) and one nipple drinker (out of 11 drinkers) from 0912 to 1012 on 35 days of bird age in one experimental room. Broilers spent overall 336.7 bird-min at feeder and 34.3 bird-min at drinker, respectively, within the hour. At any time during this 1-h period, 2 to 11 broilers stayed at feeder and 0 to 3 broilers were at drinker. Supplementary Figure S7 shows the distribution of bird numbers simultaneously presenting at feeder and drinker in the hour. The tube feeder was designed with 14 feeding slots which, however, were never occupied by 14 broilers. Eighty eight percent (88%) of the time the feeder was used by five to eight broilers (Supplementary Figure S7a). The events of three birds being at drinker simultaneously accounted for less than 0.1% of the time. The scenario of no birds using the drinker took up most of the time (49.1%) in this hour (Supplementary Figure S7b). Figure 7b and 7c show seven randomly selected birds at feeder and drinker, respectively. During this hour, different birds exhibited different feeding and drinking patterns. The average and 95% confidence interval for TS were 11.8±6.1 min at feeder and 2.8±1.7 min at drinker, and 7.2 to 16.3 min at feeder and 1.6 to 4.1 min at drinker for these seven individual birds during this hour.

## Discussion

### Max reading distances of tags

With five extra tags, the SD of MRD for the same tags was 1 to 2 cm, whereas the SD of MRD for different tags was 7 to 8 cm. Therefore, the tag variations were mainly caused by different tags rather than by measurement differences. During the test, the zip-tie end of the tag (Supplementary Figure S1) was held by a hand to minimize the hand effect on the readings. Max reading distances of the five tags held by wooden sticks were compared with those held by a hand, but little difference was noticed between these two methods. Therefore, the hand had little effect on the results. Significant variations of commercial tags with the same manufacturing specification indicated the user to verify the tags before using them for animal tests. Common method (e.g. IQR method in this case) was recommended to select the tags with similar sensitivities, which could minimize reading bias caused by tag variations.

During the test, a tag was placed to the neck of each bird (Supplementary Figure S2). The broilers felt uncomfortable at the first 3 h after the tag placement. Then they got used to the tags and could eat and drink normally based on daily inspection by the caretaker. The collar size required adjustments to avoid discomfort (e.g. panting, choking, etc.) as the broilers grew, but it was doable for the lab test.

### Duration of intermittent withdrawal from a feeder/drinker in a continuous feeding/drinking event

The 20-s threshold was used to fill the time gaps in the RFID readings when characterizing feeding and drinking behaviors. Li *et al*. (2017) used a time threshold of 30 s for feeding and nesting behaviors of laying hens. A shorter time threshold of 20 s was identified in this study, possibly because broilers were motivated to eat fast, thus a reduced swallowing time compared to laying hens (Bizeray *et al*., 2002a).

### Accuracy of the ultra-high frequency radio frequency identification system

Accuracies for the vertical antenna placements were relatively low because the drinker antenna failed to register drinking birds outside its detection range. Based on our measurement, the distance between the tags attached to the bird necks and the vertically placed drinker antenna could be greater than 8 cm which was beyond the detection range (6 cm) of the antenna. Tilting the support bracket and the antenna toward the drinking broilers profoundly increased the accuracy of the UHF-RFID system. Misidentification of drinking broilers may occur when a bird approached the drinker from sides of the antenna, which was more likely to happen when multiple birds try to drink simultaneously at the same drinker. Tags may occasionally turn to the back side of the bird necks which resulted in failure in tag detection by the drinker antenna and should be avoided through regularly checking of the tags.

The sensitivity, specificity, accuracy, and precision as described in Adrion *et al*. (2018) for evaluating the system performance, are not relevant in our study because of two reasons. First, we were working with a large group of broilers (60 birds/pen) which made it difficult/impossible to visually identify individual birds and compare them with RFID data. Second, the tube feeder/drinker and our RFID system were designed for a group of broilers eating/drinking simultaneously, rather than individual feeder/drinker space like for pigs reported by Adrion *et al*. (2018). Therefore, the accuracy in this study referred to Li *et al*. (2017). It reflected the chance of the RFID system to recognize the correct number of birds at feeder/drinker.

Li *et al*. (2017) developed an UHF-RFID system in the enriched colony housing system for detecting the feeding and nesting behaviors of individual laying hens. The accuracies of the UHF-RFID system were 92.1±6.4% for feeding behaviors and 91.4±1.7% for nesting behaviors. Sales *et al*. (2015) detected hen transitions between environmentally controlled chambers using a RFID system, and reported that the accuracies were 91.0±2.6% for total TS in chambers and 85.8±8.0% for TS per visit. Thurner *et al*. (2008) developed the high frequency transponder system to register the laying behaviors of individual hens in the floor rearing system. Of 374 visits to the nest boxes, 89.8% were correctly registered. The accuracies of the UHF-RFID system developed in this study (92.5% to 99.0% for feeding behaviors; 93.7% to 94.7% for drinking behaviors) were comparable or higher than those reported previously. The set up of the UHF-RFID system worked for the broilers from 28 to 35 days of age. We believe the system will work for older broilers (up to nine weeks old) as well because the electromagnetic fields of the antennas may well cover the locations of tags attached to older feeding/drinking broilers through proper system adjustments. For younger broilers (e.g. <one week old), the system may not work as it is hard to attach tags to the birds. Additional system validation is recommended for broilers at other ages.

### Continuous behavioral monitoring by the ultra-high frequency radio frequency identification system

Based on the 1-h sample data, the capacities of the feeder and drinker were not fully utilized. Compared to other studies that recommended 54 to 75 birds/feeder (Newberry and Hall, 1990; Dozier *et al*., 2005) and 7 to 13 birds/drinker (Bizeray *et al*., 2002b; Dozier *et al*., 2005), the broilers were provided with more feeding and drinking resources in this study, i.e. 30 birds/feeder and 6 birds/drinker. In this study, not all feeders and drinkers in the experimental room were mounted with RFID antennas. This setup served well the major objective of this study, that is, validation of the accuracy of the UHF-RFID system. When diurnal feeding and drinking rhythms of individual broilers are of interest, it can be readily achieved by expanding the UHF-RFID system to all feeders and drinkers. Overall, the UHF-RFID system is a useful tool for investigating individual broiler behaviors and resource allocation in group rearing settings.

## Conclusions

An UHF-RFID system for monitoring feeding and drinking behaviors of individual group-housed broilers was developed and tested. Tag sensitivity and modified electromagnetic fields of the feeder and drinker antennas were investigated. The UHF-RFID system was validated in two experimental rooms with 120 broilers. The results show significant sensitivity variations among tags, thus the tags with similar sensitivities should be selected for animal tests. The electromagnetic fields at the corners of the feeder antenna (40×40 cm) could be effectively shielded by covering the antenna using one layer of the stainless steel sheet with a 36-cm-diameter center opening. Protective plastic wraps, a carton box, a fully loaded feeder had little effect on the electromagnetic field of the feeder antenna. The accuracies of the UHF-RFID system for determining IBN and TS were 92.5±4.2% and 99.0±1.2% for the feeder, and 94.7±4.2% and 93.7±6.9% for the drinker, respectively. Drinker antennas required adjustment to minimize distance to the tagged broilers while drinking in order to achieve greater accuracy. The UHF-RIFD system successfully registered feeding and drinking behaviors of individual broilers in group settings with high accuracy, and thus is a useful tool for investigating the impacts of resource allocations and management practices on broiler behaviors.

## Software and data repository resources

None of the data were deposited in an official repository.

## Supplementary material

For supplementary material accompanying this paper visit https://doi.org/10.1017/S1751731118003440. click here to view supplementary material
