# Supplementary material for: An ultra-high frequency radio frequency identification system for studying individual feeding and drinking behaviors of group-housed broilers
Source: Animal. 2019 Jan 11;13(9):2060–9. doi: 10.1017/S1751731118003440 (PMC6700711; doi:10.1017/S1751731118003440)
Supplement: Supplementary file 1 [file S1751731118003440sup001.docx]

An Ultra-High Frequency Radio Frequency Identification System for Studying Individual Feeding and Drinking Behaviors of Group-Housed Broilers

Guoming Li, Yang Zhao, Rhet Hailey, Nan Zhang, Yuanji Liang, Joseph L. Purswell

**Supplementary material for Animal journal**

**Table S1** *Power settings and center opening diameters of stainless steel sheets for testing shielding effects at the corners of the feeder antenna in terms of Ross×Ross 708 broilers*

| Diameter of center opening (cm) | Power setting (W) |
| --- | --- |
| 46 | 1.0 |
| 43 | 1.0 |
| 41 | 1.0 |
| 38 | 1.0 |
| 36 | 1.0 |
| 36×2* | 1.0 |
| 36 | 0.8 |
| 36 | 0.6 |
| 36 | 0.5 |

*Two layers of steel sheets with 36-cm center opening

**Table S2** *Scenarios for testing effects on the electromagnetic field of the feeder antenna by a single layer of protective plastic wrap, an empty carton box and a fully loaded feeder in terms of Ross×Ross 708 broilers*

| Scenario | Items |
| --- | --- |
| 1 | Without plastic wrap, carton box, and feeder |
| 2 | With plastic wrap only |
| 3 | With plastic wrap and carton box |
| 4 | With plastic wrap, carton, and fully loaded feeder |

**Table S3** *The mean maximum reading distances (MRDs) of radio frequency identification tags at corners of the feeder antenna shielded by stainless steel sheet with different opening sizes at four power settings for Ross×Ross 708 broilers*

| Diameter of center opening and power setting | n | Mean (cm) | Standard deviation (cm) |
| --- | --- | --- | --- |
| 46 cm + 1.0 W | 4 | 79.9^a^ | 2.2 |
| 43 cm + 1.0 W | 4 | 78.7^a^ | 0.8 |
| 41 cm + 1.0 W | 4 | 78.7^a^ | 0.3 |
| 38 cm + 1.0 W | 4 | 62.4^b^ | 2.4 |
| 36 cm + 1.0 W | 4 | 31.1^c^ | 0.9 |
| 36 cm × 2* + 1.0 W | 4 | 30.5^c^ | 0.3 |
| 36 cm + 0.8 W | 4 | 13.5^d^ | 1.3 |
| 36 cm + 0.6 W | 4 | 5.1^e^ | 1.1 |
| 36 cm + 0.5 W | 4 | 2.3^f^ | 0.2 |
| P-value | <0.0001 | | |
| Root mean square error | 0.46 | | |

^a, b, c, d, e, f^ Values within a column with different superscripts differ significantly at P<0.05 (PROC GLM, LSD test). * Two layers of steel sheets with 36-cm-diameter center opening.

**Table S4** *The maximum reading distance of radio frequency identification tags above a feeder antenna with or without protective plastic wrap, carton box, and feed/feeder for Ross×Ross 708 broilers*

| Scenario | n | Mean (cm) | Standard deviation (cm) |
| --- | --- | --- | --- |
| 1 | 4 | 82.3^a^ | 0.9 |
| 2 | 4 | 81.9^a^ | 1.2 |
| 3 | 4 | 81.5^a^ | 2.5 |
| 4 | 4 | 81.3^a^ | 1.2 |
| P-value | 0.47 | | |
| Root mean square error (RMSE) | 0.25 | | |

^a^ Values within a column with different superscripts differ significantly at P<0.05 (PROC GLM, LSD test). Scenario 1 = Without plastic wrap, carton box, and feeder; Scenario 2 = With plastic wrap only; Scenario 3 = With plastic wrap and carton box; Scenario 4 = With plastic wrap, carton, and fully loaded feeder.

**Table S5** *The maximum reading distance of radio frequency identification tags above a drinker antenna with or without protective plastic wrap for Ross×Ross 708 broilers*

| Scenario | n | Mean (cm) | Standard deviation (cm) |
| --- | --- | --- | --- |
| 1 | 4 | 6.3^a^ | 2.4 |
| 2 | 4 | 6.1^a^ | 1.5 |
| P-value | 0.34 | | |
| Root mean square error (RMSE) | 0.19 | | |

^a^ Values within a column with different superscripts differ significantly at P<0.05 (PROC GLM, LSD test). Scenario 1 = Without plastic wrap; Scenario 2 = With plastic wrap.


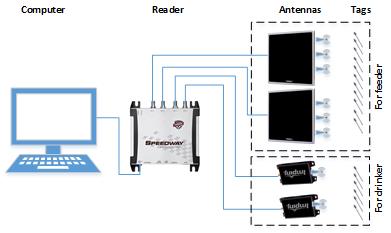


**Figure S1** Schematic illustration of the ultra-high frequency radio frequency identification system and its components for Ross×Ross 708 broilers


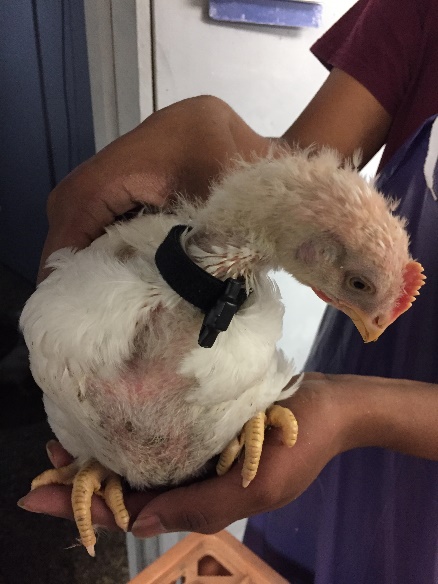


**Figure S2** A Ross×Ross 708 broiler with a radio freqeuncy identification tag on its neck


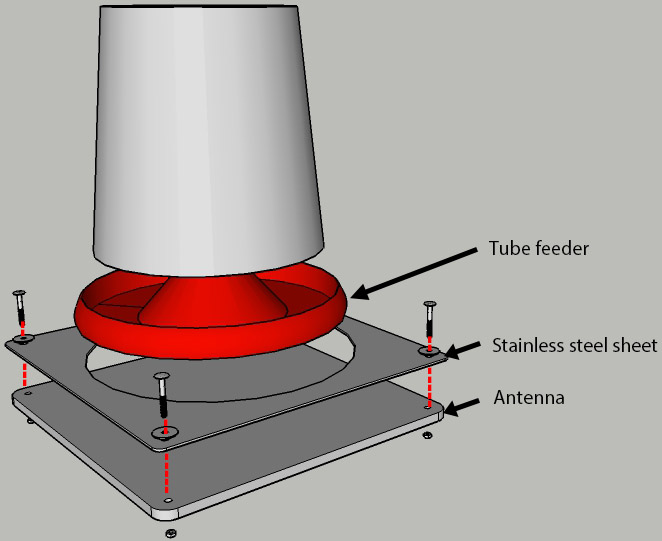


**Figure S3** Schematic illustration of system setup for testing shielding effect of electromagnetic field at the corners of a feeder antenna using a stainless steel sheet with a center opening for Ross×Ross 708 broilers

| 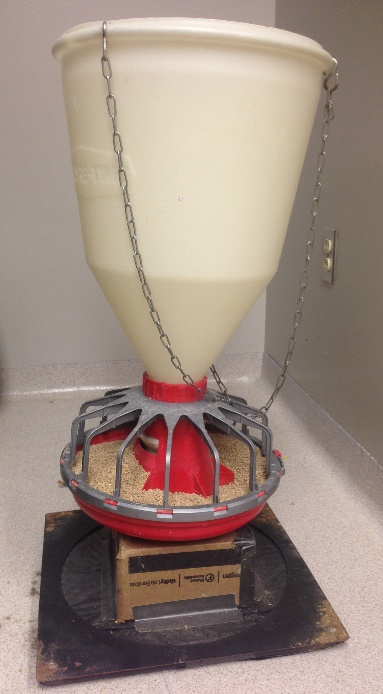 | 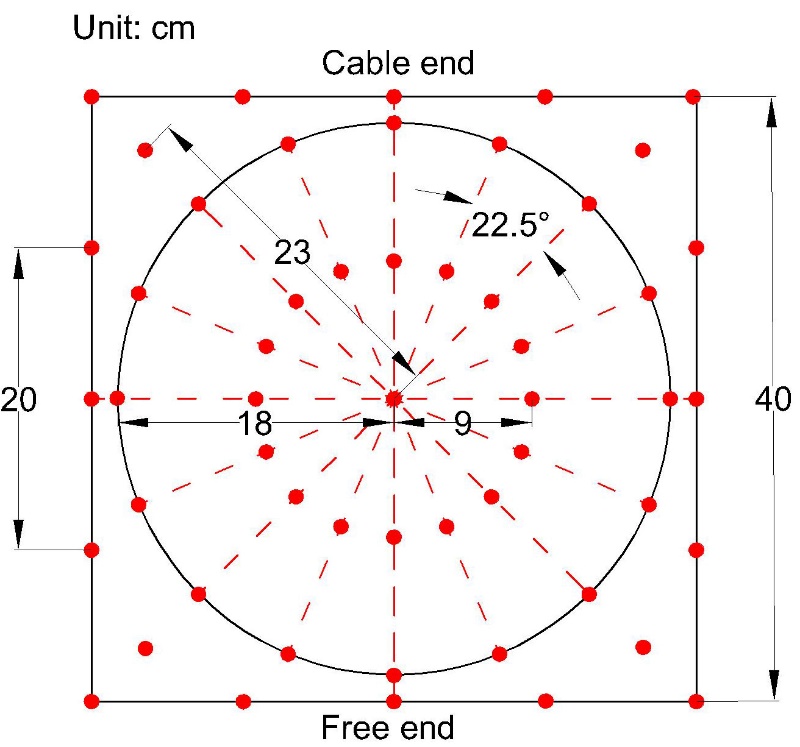 |
| --- | --- |
| (a) | (b) |

**Figure S4** System setup of electromagnetic field test for the feeder antenna (a), and top view of the antenna and test points indicated by red dots (b) for Ross×Ross 708 broilers

| 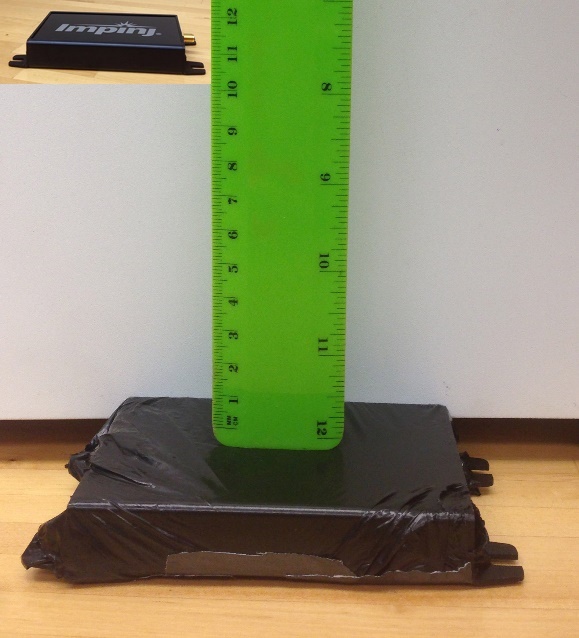 | 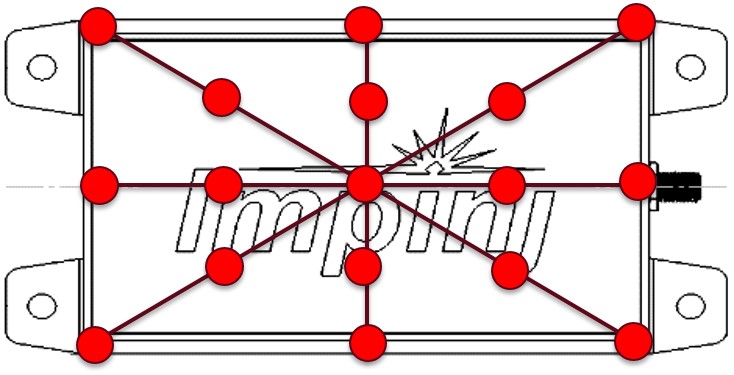 |
| --- | --- |
| (a) | (b) |

**Figure S5** System setup of electromagnetic field test for the drinker antenna (a), and top view of the antenna and test points indicated by red dots (b) in terms of Ross×Ross 708 broilers

| 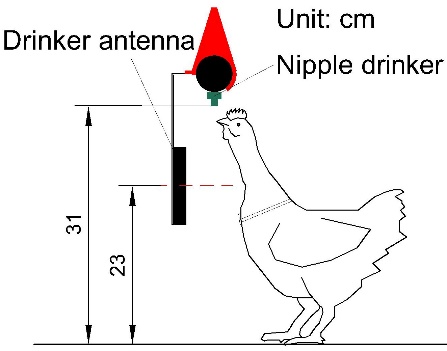 | 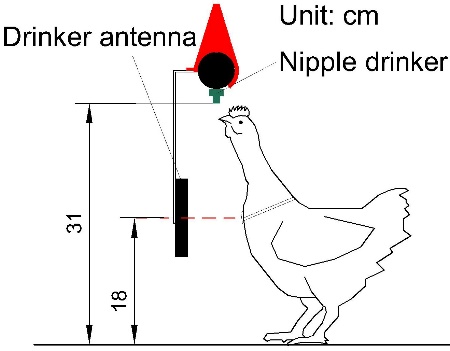 | 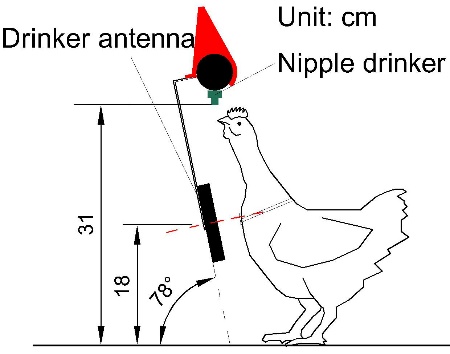 |
| --- | --- | --- |
| （a） | (b) | (c) |

**Figure S6** Placements of antennas in validation tests for drinking behaviors of Ross×Ross 708 broilers: (a) vertical placement at 23 cm height; (b) vertical placement at 18 cm height; (c) tilting placement at 18 cm height

| 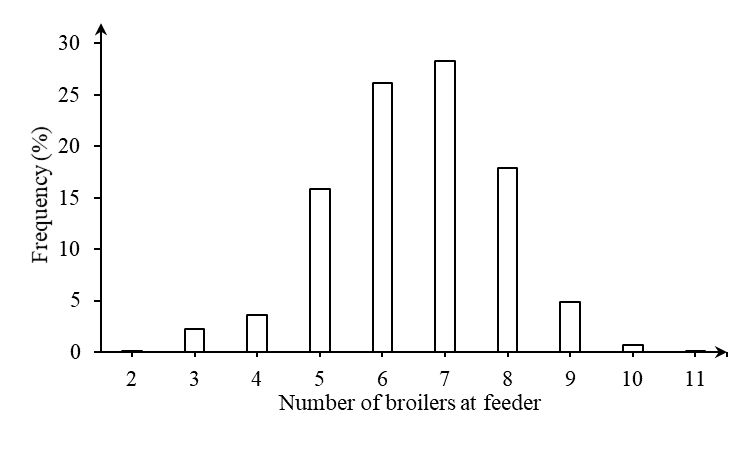 | 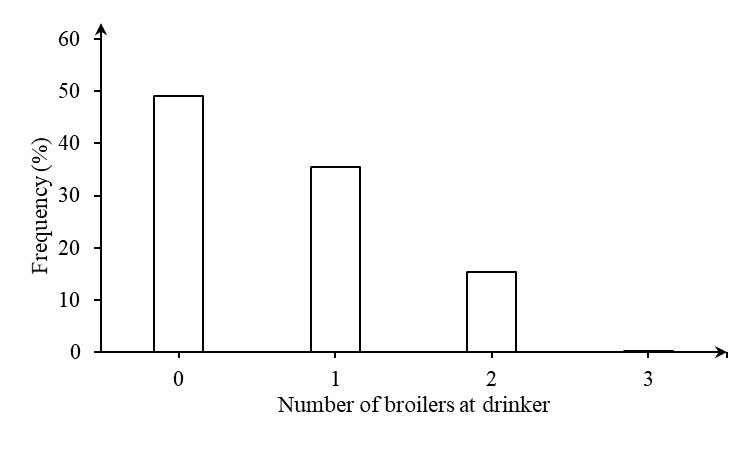 |
| --- | --- |
| (a) | (b) |

**Figure S7** Histogram of feeder (a) and drinker (b) occupancy by Ross×Ross 708 broilers (0912 to 1012 on 35 days of age)

**Material S1** GLM procedure and model description

A one-way generalized linear model (GLM) procedure was introduced to analyze the shielding effect and add-ons effect on max reading distance (MRD). The model is defined as

$Y_{ij}=\mu+\alpha_{i}+\varepsilon_{ij}$ (S2-1)

Where

$Y_{ij}$ is the measured max reading distance

$\mu$ is the overall mean

$\alpha_{i}$ is the main effect of diameters of opening and power, or the main effect of the add-ons

$\varepsilon_{ij}$ is the random error for the model

**Material S2** SAS code

**PROC** **IMPORT** OUT= WORK.Scenarios

DATAFILE= "path of data file"

DBMS=EXCEL REPLACE;

RANGE="Size$";

GETNAMES=YES;

MIXED=NO;

SCANTEXT=YES;

USEDATE=YES;

SCANTIME=YES;

**RUN**;

**PROC** **GLM** DATA= Scenarios;

CLASS number;

MODEL mrd=number;

MEANS number/LSD CLDIFF;

**RUN**;

**PROC** **MEANS** DATA=Scemarios MEAN STD;

CLASS number;

VAR mrd;

**RUN**;
